# Reporting bias in medical research - a narrative review

**DOI:** 10.1186/1745-6215-11-37

**Published:** 2010-04-13

**Authors:** Natalie McGauran, Beate Wieseler, Julia Kreis, Yvonne-Beatrice Schüler, Heike Kölsch, Thomas Kaiser

**Affiliations:** 1 Institute for Quality and Efficiency in Health Care, Dillenburger Str 27, 51105 Cologne, Germany

## Abstract

Reporting bias represents a major problem in the assessment of health care interventions. Several prominent cases have been described in the literature, for example, in the reporting of trials of antidepressants, Class I anti-arrhythmic drugs, and selective COX-2 inhibitors. The aim of this narrative review is to gain an overview of reporting bias in the medical literature, focussing on publication bias and selective outcome reporting. We explore whether these types of bias have been shown in areas beyond the well-known cases noted above, in order to gain an impression of how widespread the problem is. For this purpose, we screened relevant articles on reporting bias that had previously been obtained by the German Institute for Quality and Efficiency in Health Care in the context of its health technology assessment reports and other research work, together with the reference lists of these articles.

We identified reporting bias in 40 indications comprising around 50 different pharmacological, surgical (e.g. vacuum-assisted closure therapy), diagnostic (e.g. ultrasound), and preventive (e.g. cancer vaccines) interventions. Regarding pharmacological interventions, cases of reporting bias were, for example, identified in the treatment of the following conditions: depression, bipolar disorder, schizophrenia, anxiety disorder, attention-deficit hyperactivity disorder, Alzheimer's disease, pain, migraine, cardiovascular disease, gastric ulcers, irritable bowel syndrome, urinary incontinence, atopic dermatitis, diabetes mellitus type 2, hypercholesterolaemia, thyroid disorders, menopausal symptoms, various types of cancer (e.g. ovarian cancer and melanoma), various types of infections (e.g. HIV, influenza and Hepatitis B), and acute trauma. Many cases involved the withholding of study data by manufacturers and regulatory agencies or the active attempt by manufacturers to suppress publication. The ascertained effects of reporting bias included the overestimation of efficacy and the underestimation of safety risks of interventions.

In conclusion, reporting bias is a widespread phenomenon in the medical literature. Mandatory prospective registration of trials and public access to study data via results databases need to be introduced on a worldwide scale. This will allow for an independent review of research data, help fulfil ethical obligations towards patients, and ensure a basis for fully-informed decision making in the health care system.

## Background

The reporting of research findings may depend on the nature and direction of results, which is referred to as "reporting bias" [1,2]. For example, studies in which interventions are shown to be ineffective are sometimes not published, meaning that only a subset of the relevant evidence on a topic may be available [1,2]. Various types of reporting bias exist (Table 1), including publication bias and outcome reporting bias, which concern bias from missing outcome data on 2 levels: the study level, i.e. "non-publication due to lack of submission or rejection of study reports", and the outcome level, i.e. "the selective non-reporting of outcomes within published studies" [3].

**Table 1:** Definitions of some types of reporting bias^1^

Type of reporting bias	Definition
Publication bias	The *publication *or *non-publication *of research findings, depending on the nature and direction of the results
Time lag bias	The *rapid *or *delayed *publication of research findings, depending on the nature and direction of the results
Multiple (duplicate) publication bias	The *multiple *or *singular *publication of research findings, depending on the nature and direction of the results
Location bias	The publication of research findings in journals with different *ease of access *or *levels of indexing *in standard databases, depending on the nature and direction of results
Citation bias	The *citation *or *non-citation *of research findings, depending on the nature and direction of the results
Language bias	The publication of research findings *in a particular language*, depending on the nature and direction of the results
Outcome reporting bias	The *selective reporting *of some outcomes but not others, depending on the nature and direction of the results

^1^Table 10.1.a, Chapter 10 of the Cochrane Handbook for Systematic Reviews of Interventions [2]. © The Cochrane Collaboration. Reproduced with permission.

### Reporting bias on a study level

Results of clinical research are largely underreported or reported with delay. Various analyses of research protocols submitted to institutional review boards and research ethics committees in Europe, the United States, and Australia found that on average, only about half of the protocols had been published, with higher publication rates in Anglo-Saxon countries [4-10].

Similar analyses have been performed of trials submitted to regulatory authorities: a cohort study of trials supporting new drugs approved by the Food and Drug Administration (FDA) identified over 900 trials of 90 new drugs in FDA reviews; only 43% of the trials were published [11]. Wide variations in publication rates have been shown for specific indications [12-16]. The selective submission of clinical trials with positive outcomes to regulatory authorities has also been described [17]. Even if trials are published, the time lapse until publication may be substantial [8,18,19].

There is no simple classification of a clinical trial into "published" or "unpublished", as varying degrees of publication exist. These range from full-text publications in peer-reviewed journals that are easily identifiable through a search in bibliographic databases, to study information entered in trial registries, so-called grey literature (e.g. abstracts and working papers), and data on file in drug companies and regulatory agencies, which may or may not be provided to health technology assessment (HTA) agencies or other researchers after being requested. If such data are transmitted, they may then be fully published or not (e.g. the German Institute for Quality and Efficiency in Health Care [Institut für Qualität und Wirtschaftlichkeit im Gesundheitswesen, IQWiG] publishes all data used in its assessment reports [20], whereas the UK National Institute for Clinical Excellence [NICE] may accept "commercial in confidence" data [21]).

Even if studies are presented at meetings, this does not necessarily mean subsequent full publication: an analysis of nearly 30,000 meeting abstracts from various disciplines found a publication rate of 63% for randomized or controlled clinical trials [22].

### Reporting bias on an outcome level

Selective reporting within a study may involve (a) selective reporting of analyses or (b) selective reporting of outcomes. This may include, for example, the reporting of (a) per-protocol (PP) versus intention-to-treat (ITT) analyses or adjusted versus unadjusted analyses, and (b) outcomes from different time points or statistically significant versus non-significant outcomes [3,23].

Various reviews have found extensive selective reporting in study publications [3,14,24-28]. For example, comparisons of publications with study protocols have shown that primary outcomes had been newly introduced, omitted, or changed in about 40% to 60% of cases [3,24]. Selective reporting particularly concerns the underreporting of adverse events [12,29-32]. For example, an analysis of 192 randomized drug trials in various indications showed that only 46% of publications stated the frequency of specific reasons for treatment discontinuation due to toxicity [29]. Outcomes are not only selectively reported, but negative results are reported in a positive manner and conclusions are often not supported by results data [16,26,33-35]. For instance, a comparison of study characteristics reported in FDA reviews of New Drug Applications (NDAs) with those reported in publications found that 9 of 99 conclusions had been changed in the publications, all in favour of the new drug [26].

### Factors associated with reporting bias

#### Characteristics of published studies

The fact that studies with positive or favourable results are more likely to be published than those with negative or unfavourable results was already addressed in the 1950s [36], and has since been widely confirmed [3,6-8,14,37-40]. Studies with positive or favourable results have been associated with various other factors such as faster publication [8,18,19,37], publication in higher impact factor journals [7,41], a greater number of publications [7] (including covert duplicate publications [42]), more frequent citation [43-45], and more likely publication in English [46].

Several other factors have been linked to successful publication, for example, methodological quality [47], study type [47], sample size [5,7,48], multicentre status [5,6,41], and non-commercial funding [5,6,49,50]. However, for some factors, these associations are inconsistent [6,37].

#### Submission and rejection of studies

One of the main reasons for the non-publication of negative studies seems to be the non-submission of manuscripts by investigators, not the rejection of manuscripts by medical journals. A follow-up of studies approved by US institutional review boards showed that only 6 of 124 unpublished studies had actually been rejected for publication [6]. A prospective cohort study of 745 manuscripts submitted to JAMA showed no statistically significant difference in publication rates between studies with positive and those with negative results [51], which has been confirmed by further analyses of other journals [47,52]. Author surveys have shown that the most common reasons for not submitting papers were negative results and a lack of interest, time, or other resources [39-41,53].

#### The role of the pharmaceutical industry

An association has been shown between industry sponsorship or industry affiliation of authors and positive research outcomes and conclusions, both in publications of primary studies and in systematic reviews [49,54-63]. For example, in a systematic review of the scope and impact of financial conflicts of interest in biomedical research, an aggregation of the results of 8 analyses of the relation between industry sponsorship and outcomes showed a statistically significant association between industry sponsorship and pro-industry conclusions [55]. A comparison of the methodological quality and conclusions in Cochrane reviews with those in industry-supported meta-analyses found that the latter were less transparent, less critical of methodological limitations of the included trials, and drew more favourable conclusions [57]. In addition, publication constraints and active attempts to prevent publication have been identified in industry-sponsored research [55,64-68]. Other aspects of industry involvement, such as design bias, are beyond the scope of this paper.

## Rationale, aim and procedure

IQWiG produces HTA reports of drug and non-drug interventions for the decision-making body of the statutory health care funds, the Federal Joint Committee. The process of report production includes requesting information on published and unpublished studies from manufacturers; unfortunately, compliance by manufacturers is inconsistent, as recently shown in the attempted concealment of studies on antidepressants [69]. Reporting bias in antidepressant research has been shown before [16,70]; other well-known cases include Class I anti-arrhythmic drugs [71,72] and selective COX-2 inhibitors [73,74].

The aim of this narrative review was to gain an overview of reporting bias in the medical literature, focussing on publication bias and selective outcome reporting. We wished to explore whether this type of bias has been shown in areas beyond the well-known cases noted above, in order to obtain an impression of how widespread this problem is. The review was based on the screening of full-text publications on reporting bias that had either been obtained by the Institute in the context of its HTA reports and other research work or were identified by the screening of the reference lists of the on-site publications. The retrieved examples were organized according to indications and interventions. We also discuss the effects of reporting bias, as well as the measures that have been implemented to solve this problem.

The term "reporting bias" traditionally refers to the reporting of clinical trials and other types of studies; if one extends this term beyond experimental settings, for example, to the withholding of information on any beneficial medical innovation, then an early example of reporting bias was noted by Rosenberg in his article "Secrecy in medical research", which describes the invention of the obstetrical forceps. This device was developed by the Chamberlen brothers in Europe in the 17th century; however, it was kept secret for commercial reasons for 3 generations and as a result, many women and neonates died during childbirth [75]. In the context of our paper, we also considered this extended definition of reporting bias.

## Findings

We identified reporting bias in 40 indications comprising around 50 different interventions. Examples were found in various sources, e.g. journal articles of published versus unpublished data, reviews of reporting bias, editorials, letters to the editor, newspaper reports, expert and government reports, books, and online sources. The following text summarizes the information presented in these examples. More details and references to the background literature are included in Additional file 1: Table S2.

### Mental and behavioural disorders

Reporting bias is common in psychiatric research (see below). This also includes industry-sponsorship bias [76-82].

#### Depression

Turner et al compared FDA reviews of antidepressant trials including over 12,000 patients with the matching publications and found that 37 out of 38 trials viewed as positive by the FDA were published [16]. Of the 36 trials having negative or questionable results according to the FDA, 22 were unpublished and 11 of the 14 published studies conveyed a positive outcome. According to the publications, 94% of the trials had positive results, which was in contrast to the proportion reported by the FDA (51%). The overall increase in effect size in the published trials was 32%. In a meta-analysis of data from antidepressant trials submitted to the FDA, Kirsch et al requested data on 6 antidepressants from the FDA under the Freedom of Information Act. However, the FDA did not disclose relevant data from 9 of 47 trials, all of which failed to show a statistically significant benefit over placebo. Data from 4 of these trials were available on the GlaxoSmithKline (GSK) website. In total, the missing data represented 38% of patients in sertraline trials and 23% of patients in citalopram trials. The analysis of trials investigating the 4 remaining antidepressants showed that drug-placebo differences in antidepressant efficacy were relatively small, even for severely depressed patients [83].

##### Selective serotonin reuptake inhibitors (SSRIs)

One of the biggest controversies surrounding unpublished data was the withholding of efficacy and safety data from SSRI trials. In a lawsuit launched by the Attorney General of the State of New York it was alleged that GSK had published positive information about the paediatric use of paroxetine in major depressive disorder (MDD), but had concealed negative safety and efficacy data [84]. The company had conducted at least 5 trials on the off-label use of paroxetine in children and adolescents but published only one, which showed mixed results for efficacy. The results of the other trials, which did not demonstrate efficacy and suggested a possible increased risk of suicidality, were suppressed [84]. As part of a legal settlement, GSK agreed to establish an online clinical trials registry containing results summaries for all GSK-sponsored studies conducted after a set date [85,86].

Whittington et al performed a systematic review of published versus unpublished data on SSRIs in childhood depression. While published data indicated a favourable risk-benefit profile for some SSRIs, the inclusion of unpublished data indicated a potentially unfavourable risk-benefit profile for all SSRIs investigated except fluoxetine [70].

##### Newer antidepressants

IQWiG published the preliminary results of an HTA report on reboxetine, a selective norepinephrine reuptake inhibitor, and other antidepressants. At least 4600 patients had participated in 16 reboxetine trials, but the majority of data were unpublished. Despite a request for information the manufacturer Pfizer refused to provide these data. Only data on about 1600 patients were analysable and IQWiG concluded that due to the risk of publication bias, no statement on the benefit or harm of reboxetine could be made [69,87]. The preliminary HTA report mentioned above also included an assessment of mirtazapine, a noradrenergic and specific serotonergic antidepressant. Four potentially relevant trials were identified in addition to 27 trials included in the assessment, but the manufacturer Essex Pharma did not provide the study reports. Regarding the other trials, the manufacturer did not send the complete study reports, so the full analyses were not available. IQWiG concluded that the results of the assessment of mirtazapine may have been biased by unpublished data [69,87]. After the behaviour of Pfizer and Essex Pharma had been widely publicized, the companies provided the majority of study reports for the final HTA report. The preliminary report's conclusion on the effects of mirtazapine was not affected by the additional data. For reboxetine, the analysis of the published and unpublished data changed the conclusion from "no statement possible" to "no benefit proven" [88].

#### Bipolar disorder

##### Lamotrigine

A review by Nassir Ghaemi et al of data on lamotrigine in bipolar disorder provided on the GSK website showed that data from negative trials were available on the website but that the studies had not been published in detail or publications emphasized positive secondary outcomes instead of negative primary ones. Outside of the primary area of efficacy (prophylaxis of mood episodes), the drug showed very limited efficacy in indications such as acute bipolar depression, for which clinicians were supporting its use [35].

##### Gabapentin

Gabapentin, a GABA analogue, was approved by the FDA in 1993 for a certain type of epilepsy, and in 2002 for postherpetic neuralgia. As of February 1996, 83% of gabapentin use was for epilepsy, and 17% for off-label indications (see the expert report by Abramson [89]). As the result of a comprehensive marketing campaign by Pfizer, the number of patients in the US taking gabapentin rose from about 430,000 to nearly 6 million between 1996 and 2001; this increase was solely due to off-label use for indications, including bipolar disorder. As of September 2001, 93.5% of gabapentin use was for off-label indications [89]. In a further expert report, Dickersin noted "extensive evidence of reporting bias" [34], which she further analysed in a recent publication with Vedula et al [90]. Concerning the trials of gabapentin for bipolar disorders, 2 of the 3 trials (all published) were negative for the primary outcome. However, these publications showed "extensive spin and misrepresentation of data" [34].

#### Schizophrenia

##### Quetiapine

The Washington Post reported that a trial on quetiapine, an atypical antipsychotic, was "silenced" in 1997, the same year it was approved by the FDA to treat schizophrenia. The study ("Study 15") was not published. Patients taking quetiapine had shown high rates of treatment discontinuations and had experienced significant weight increases. However, data presented by the manufacturer AstraZeneca in 1999 at European and US meetings actually indicated that the drug helped psychotic patients lose weight [91].

#### Panic disorder

##### Paroxetine

Turner described an example of reporting bias in the treatment of panic disorder: according to a review article, 3 "well designed studies" had apparently shown that the controlled-release formulation of paroxetine had been effective in patients with this condition. However, according to the corresponding FDA statistical review, only one study was strongly positive, the second study was non-significant regarding the primary outcome (and marginally significant for a secondary outcome), and the third study was clearly negative [92].

Further examples of reporting bias in research on mental and behavioural disorders are included in Additional file 1: Table S2.

### Disorders of the nervous system

#### Alzheimer's disease

##### Rofecoxib

Internal company analyses and information provided by the manufacturer Merck & Co to the FDA on rofecoxib, a selective COX-2 inhibitor, were released during litigation procedures. The documents referred to trials investigating the effects of rofecoxib on the occurrence or progression of Alzheimer's disease. Psaty and Kronmal performed a review of these documents and 2 trial publications and showed that, although presenting mortality data, the publications had not included analyses or statistical tests of these data and both had concluded that regarding safety, rofecoxib was "well tolerated". In contrast, in April 2001, Merck's internal ITT analyses of pooled data from these 2 trials showed a significant increase in total mortality. However, this information was neither disclosed to the FDA nor published in a timely fashion [74]. Rofecoxib was taken off the market by Merck in 2004 [93], among allegations that the company had been aware of the safety risks since 2000 [73].

#### Acute pain

##### Valdecoxib

In their article "An untold story?", Lenzer and Brownlee reported the case of valdecoxib, another selective COX-2 inhibitor withdrawn from the market due to cardiovascular concerns [94,95]. In 2001, the manufacturer Pfizer had applied for approval in 4 indications, including acute pain. The application for acute pain was rejected and some of the information about the corresponding trials removed from the FDA website for confidentiality reasons. Further examples of reporting bias in research on pain are presented in Additional file 1: Table S2.

#### Migraine

##### Gabapentin

According to the expert report by Dickersin, all 3 trials on gabapentin for migraine showed negative results for the primary outcome. Substantial reporting bias was present. One trial was fully published (seemingly with a redefined primary outcome showing positive results in a subgroup of patients), one was unpublished, and preliminary (positive) results were presented for the third trial [34].

### Disorders of the circulatory system

#### Coronary heart disease (bleeding prophylaxis during bypass surgery)

##### Aprotinin

In his article on observational studies on drug safety, Hiatt reported the case of aprotinin, an antifibrinolytic drug formerly marketed to reduce bleeding during heart bypass graft surgery. In 2006, data from 2 published observational studies indicated serious concerns about the drug's safety [96]. The FDA subsequently convened an expert meeting in which the safety data presented by the manufacturer Bayer did not reveal any increased risk of fatal or nonfatal cardiovascular events. However, it turned out that Bayer had not presented additional observational data, which, according to an FDA review, indicated that aprotinin may be associated with an increased risk of death and other serious adverse events. In November 2007 Bayer suspended the worldwide marketing of aprotinin, after requests and advice from various drug regulating authorities [97].

#### Prevention of arrhythmia

##### Class I anti-arrhythmic drugs

In a clinical trial conducted in 1980, 9 out of 49 patients with suspected acute myocardial infarction treated with a class Ic anti-arrhythmic drug (lorcainide) died, versus only one patient in the placebo group; the investigators interpreted this finding as an "effect of chance" [71]. The development of lorcainide was discontinued for commercial reasons, and the results of the trial were not published until 1993. The investigators then stated that if the trial had been published earlier, it "might have provided an early warning of trouble ahead" [71]. Instead, during the 1980s, class I drugs were widely used, even though concerns as to their lack of effect were published as early as 1983 [98,99]. Further reviews and trials confirmed this suspicion, as well as an increase in mortality [100-102]. In his book "Deadly Medicine", Moore described the consequences as "America's worst drug disaster", which had "produced a death toll larger than the United States' combat losses in wars such as Korea and Vietnam" [72]. Further examples of reporting bias in research on disorders of the circulatory system are presented in Additional file 1: Table S2.

### Disorders of the digestive system

#### Irritable bowel syndrome

##### Alosetron

Barbehenn et al compared a published trial on alosetron, a 5-HT_3 _antagonist, in women with irritable bowel syndrome with data obtained from the FDA [103]. She noted that according to the graphics in the publication, which presented relative differences in pain and discomfort scores, the drug seemed effective. However, when plotting the absolute data from the FDA review, the data points were almost superimposable. After discussions with the FDA about potential serious side effects, the drug was withdrawn from the market by the manufacturer in 2000, but reapproved with restrictions in 2002 [104]. A further example of reporting bias in research on disorders of the digestive system is presented in Additional file 1: Table S2.

### Disorders of the genitourinary system/Perinatal medicine

#### Urinary incontinence

##### Duloxetine

Lenzer and Brownlee also reported cases of suicide in a trial investigating the selective serotonin and noradrenalin reuptake inhibitor duloxetine for a new indication, urinary incontinence in women. However, the FDA refused to release data on these cases, citing trade secrecy laws. These laws "permit companies to withhold all information, even deaths, about drugs that do not win approval for a new indication, even when the drug is already on the market for other indications" [94]. Two examples of reporting bias in perinatal research are presented in Additional file 1: Table S2.

### Disorders of the musculoskeletal system

#### Osteoarthritis

##### Rofecoxib

In 2000, a trial comparing upper gastrointestinal toxicity of rofecoxib, a selective COX-2 inhibitor, and naproxen in over 8000 patients with rheumatoid arthritis reported that rofecoxib was associated with significantly fewer clinically important upper gastrointestinal events. The significantly lower myocardial infarction rate in the naproxen group was attributed to a cardioprotective effect of naproxen (VIGOR trial, [105]). Concerns about the risk of selective COX-2-inhibitor-related cardiovascular events were raised as early as 2001 [106], and in 2002, an analysis including previously unpublished data from FDA reports of the VIGOR trial showed a statistically significant increase of serious cardiovascular thrombotic events in patients using rofecoxib [107].

##### Celecoxib

In their article on access to pharmaceutical data at the FDA, Lurie and Zieve presented the example of the selective COX-2 inhibitor celecoxib: in a journal publication of a trial investigating the gastrointestinal toxicity with celecoxib versus other pain medications, the study authors concluded that the drug was associated with a lower incidence of gastrointestinal ulcers after 6 months of therapy [108,109]. However, they failed to disclose that at the time of publication they had already received data for the full study period (12 months), which showed no advantage over the comparator drugs for the above outcome [109].

### Disorders of the skin

#### Atopic dermatitis

##### Evening primrose oil

In his editorial "Evening primrose oil for atopic dermatitis - Time to say goodnight", Williams reported that he and his colleague, who had performed an individual patient meta-analysis of evening primrose oil for atopic dermatitis commissioned by the UK Department of Health, were not given permission to publish their report, which included 10 previously unpublished studies. After submission of the report to the Department of Health, Searle, the company then responsible for product marketing, required the authors and referees to sign a written statement that the contents of the report had not been leaked. Other research had not shown convincing evidence of a benefit, and in 2002 the UK Medicines Control Agency withdrew marketing authorisation [66].

### Endocrine and metabolic disorders

#### Diabetes mellitus type 2

##### Rosiglitazone

The US cardiologist Steven Nissen commented on safety issues surrounding rosiglitazone, a thiazolidinedione used to treat type 2 diabetes. After the drug's approval, the FDA was informed in August 2005 by the manufacturer GSK that it had performed a meta-analysis of 42 randomized clinical trials of rosiglitazone, which suggested a 31% increase in the risk of ischaemic cardiovascular complications. GSK posted this finding on its website. However, neither GSK nor the FDA disseminated their findings in a broad way to the scientific community and the public [110]. The safety concerns were supported by a controversially discussed meta-analysis performed by Nissen and Wolski, who found that treatment with rosiglitazone was associated with a significantly increased risk of myocardial infarction and an increase in the risk of death from cardiovascular causes that had borderline significance [111]. More examples of reporting bias in diabetes research are presented in Additional file 1: Table S2.

#### Hypercholesterolaemia

##### Ezetimibe and simvastatin

In his article "Controversies surround heart drug study" Mitka described a trial that compared the 2 anticholesterol drugs ezetimibe and simvastatin versus simvastatin alone in patients with heterozygous familial hypercholesterolaemia [112]. No statistically significant difference between treatment groups was found for the primary outcome (mean change in the carotid intima-media thickness) after 2 years [113]. The trial, which was sponsored by Merck & Co. and Schering-Plough, was concluded in April 2006. A delay of almost 2 years in the reporting of results followed amidst allegations that the manufacturers had attempted to change the study endpoints prior to the publication of results [112]. A further case of reporting bias in research on ezetimibe is included in Additional file 1: Table S2.

##### Cerivastatin

Psaty et al conducted a review of the published literature on the statin cerivastatin and also analysed internal company documents that became available during litigation procedures [114]. In the published literature, cerivastatin was associated with a substantially higher risk of rhabdomyolysis than other statins; this particularly referred to cerivastatin-gemfibrozil combination therapy. Cerivastatin was launched in the US in 1998 by Bayer, and within 3 to 4 months, internal documents indicated there had been multiple cases of cerivastatin-gemfibrozil interactions. However, it took more than 18 months until a contraindication about the concomitant use of the 2 drugs was added to the package insert. The unpublished data available in 1999 also suggested an association between high-dose cerivastatin monotherapy and rhabdomyolysis. In 1999/2000, the company analysed FDA adverse event reporting system data, which suggested that compared with atorvastatin, cerivastatin monotherapy substantially increased the risk of rhabdomyolysis. However, these findings were not disseminated or published. Cerivastatin was removed from the market in August 2001 [114]. In the same month, the German Ministry of Health accused Bayer of withholding vital information from its federal drug agency [115].

#### Thyroid disorders

##### Levothyroxine

The Wall Street Journal reported the suppression of the results of a trial comparing the bioavailability of generic and brand-name levothyroxine products in the treatment of hypothyroidism; the investigators had concluded that the products were bioequivalent and in most cases interchangeable [116,117]. The trial was completed in 1990; over the next 7 years, the manufacturer of the brand-name product Synthroid, Boots pharmaceuticals, successfully delayed publication [65]. The manuscript was finally published in 1997.

#### Menopausal symptoms

##### Tibolone

A study investigating tibolone, a synthetic steroid, in breast-cancer patients with climacteric complaints was terminated prematurely after it was shown that this drug significantly increased the risk of cancer recurrence [118]. According to the German TV programme Frontal 21, the manufacturer (Schering-Plough, formerly NV Organon) informed regulatory authorities and ethics committees, as well as study centres and participants. However, the study results were not published until 1.5 years later [119].

### Neoplasms

Oncology is another area in which reporting bias is common [40,50,54,120-127]. A review of over 2000 oncology trials registered in ClinicalTrials.gov showed that less than 20% were available in PubMed, with substantial differences between trials sponsored by clinical trial networks and those sponsored by industry regarding both publication rates (59% vs. 6%) and the proportion of trials with positive results (50% vs. 75%) [50].

#### Ovarian cancer

##### Combination chemotherapy

In one of the earliest publications measuring the effects of reporting bias, Simes compared published oncology trials and trials identified in cancer registries that investigated the survival impact of initial alkylating agent (AA) therapy versus combination chemotherapy (CC) in advanced ovarian cancer. A meta-analysis of the published trials showed a significant survival advantage for CC; however, no such advantage was shown in the meta-analysis of registered trials [121].

#### Multiple myeloma

##### Combination chemotherapy

The above study also investigated the survival impact of AA/prednisone versus CC in multiple myeloma. The meta-analysis of published trials demonstrated a significant survival advantage for CC. A survival benefit was also shown in the registered trials; however, the estimated magnitude of the benefit was reduced [121]. A further example of reporting bias in cancer research is presented in Additional file 1: Table S2.

### Disorders of the blood

#### Thalassaemia major

##### Iron-chelation agents

In his editorial "Thyroid storm", Rennie, among other things, discussed events surrounding a US researcher who had been involved in a trial investigating the effects of an oral iron-chelation agent in patients with thalassaemia major. She had initially published an optimistic article on the effects of this agent. However, further research showed a lack of effectiveness and a potential safety risk. She had signed a confidentiality agreement but, because of her concerns, decided to break confidentiality and report her results at a meeting; the manufacturer unsuccessfully attempted to block her presentation [128].

### Bacterial, fungal, and viral infections

#### Influenza

##### Oseltamivir

The BMJ and Channel 4 News reported on the difficulties in obtaining data for an updated Cochrane review on neuraminidase inhibitors in influenza [129]. A previous analysis of oseltamivir, which was used in the prior Cochrane review [130], was based on 10 industry-sponsored trials of which only 2 had been published in peer-reviewed journals [131]. The manufacturer Roche initially declined to provide the necessary data to reproduce the analysis and then only provided a selection of files [129]. The Cochrane authors (Jefferson et al) subsequently concluded that "Evidence on the effects of oseltamivir in complications from lower respiratory tract infections, reported in our 2006 Cochrane review, may be unreliable" [132]. Roche has since agreed to provide public access to study summaries and password-protected access to the full study reports [129].

#### HIV/AIDS

##### Anti-HIV agents

Ioannidis et al identified several examples of publication bias in trials investigating medications against HIV. At least 13 trials of 6 antiviral agents including at least 3779 patients had remained unpublished for more than 3 years from the time of their meeting presentation or completion. At least 9 of these trials had negative preliminary or final results. For example, 2 large negative isoprinosine trials were unpublished, whilst a positive trial had been published in a high impact journal [33]. Further examples of reporting bias in research on infections are presented in Additional file 1: Table S2.

### Acute trauma

#### Acute spinal cord injury

##### High-dose steroids

Lenzer and Brownlee described the concerns of neurosurgeons regarding the use of high-dose steroids in patients with acute spinal cord injury. They noted that one neurosurgeon believed that several thousand patients had died as a result of this intervention; 2 surveys showed that many other neurosurgeons shared his concerns. The single available study, which had been funded by the NIH, was potentially flawed and several researchers had unsuccessfully lobbied for the release of the underlying data [94].

#### Shock

##### Human albumin infusion

In the UK Health Committee's 2004-2005 report on the influence of the pharmaceutical industry, Chalmers mentioned a systematic review of human albumin solution, which is used in the treatment of shock, e.g. in patients with burns. The results showed no evidence that albumin was helpful and suggested that this intervention may actually be harmful. Although the UK Medicines Control Agency subsequently slightly modified the labelling, it kept confidential the evidence upon which the drug had been re-licensed in 1993 [133,134].

### Vaccinations

#### HIV-1 vaccine

McCarthy reported the case of an HIV-1 vaccine study that was terminated early when no difference in efficacy between the vaccine and placebo was found. After the lead investigators refused to include a post-hoc analysis arguing that it had not been part of the study protocol and that invalid statistical methods had been used, the manufacturer, Immune Response, filed an (unsuccessful) claim seeking to prevent publication. After publication, the manufacturer filed a claim against the study's lead investigators and their universities asking for US $7-10 million in damages [135].

#### Cancer vaccines

Rosenberg provided various examples of how researchers and companies withheld information on cancer vaccines for competitive reasons; for example, researchers were asked to keep information confidential that might have prevented cancer patients from receiving ineffective or even harmful doses of a new agent [75].

### Other indications

#### Nocturnal leg cramps

##### Quinine

Man-Song-Hing et al performed a meta-analysis including unpublished individual patient data (IPD) obtained from the FDA on trials investigating quinine for the treatment of nocturnal leg cramps. They showed that the pooling only of published studies overestimated efficacy by more than 100% [136]. Further examples of reporting bias in other indications are presented in Additional file 1: Table S2.

### Further research areas

Reporting bias has also been shown in other research areas, such as genetics [137,138], effects of passive smoking [139,140] and nicotine [141,142], and effects of air pollution [143].

## Discussion

The numerous examples identified show that reporting bias concerns not only previously highlighted therapies such as antidepressants, pain medication, or cancer drugs, but affects a wide range of indications and interventions. Many cases involved the withholding of study data by manufacturers and regulatory agencies or the active attempt to suppress publication by manufacturers, which either resulted in substantial delays in publication (time-lag bias) or no publication at all.

### Limitations of the review

The review does not provide a complete overview of reporting bias in clinical research. Although our efforts to identify relevant literature went beyond the usual efforts applied in narrative reviews, the review is non-systematic and we emphasized this feature in the title. A substantial amount of relevant literature was available in-house and further relevant literature was obtained by screening reference lists. We dispensed with our initial plan to conduct a systematic review to identify cases of reporting bias, as we noticed that many cases were not identifiable by screening titles and abstracts of citations from bibliographic databases, but were "hidden" in the discussion sections of journal articles or mentioned in other sources such as newspapers, books, government reports or websites. As a search of bibliographic databases and the Internet using keywords related to reporting bias produces thousands of potentially relevant hits, we would therefore have had to obtain and read an excessive amount of full texts in order to ensure that we had not missed any examples. This was not feasible due to resource limitations. However, within the framework of a previous publication [144] we had conducted a literature search in PubMed, and some of the citations retrieved formed the basis of our literature pool for the current review. In spite of this non-systematic approach, we were able to identify dozens of cases of reporting bias in numerous indications.

Another potential limitation of the review is the validity of the sources describing cases of reporting bias. Although the majority of examples were identified in peer-reviewed journals, several cases were based on information from other sources such as newspaper articles and websites. However, we also regard these sources to be valuable as they provide a broader overview of reporting bias beyond well-known examples and also offer a starting point for more systematic research on the additional examples identified.

### Effects of reporting bias

Published evidence tends to overestimate efficacy and underestimate safety risks. The extent of misestimation is often unknown. The few identified comparisons that quantified overestimates of treatment effects in fully published versus unpublished or not fully published data showed wide variations in their results. Comparisons of pooled published versus pooled published and unpublished FDA data showed a greater treatment effect of 11% to 69% for individual antidepressants, 32% for the class of antidepressants [16], and over 100% for an agent to treat nocturnal leg cramps [136]. In addition, published studies have shown a 9% to 15% greater treatment effect than grey literature studies [145,146]. Thus, the conclusions of systematic reviews and meta-analyses based on published evidence alone may be misleading [5,7,38]. This is a serious concern as these documents are being used increasingly to support decision making in the health care system. Reporting bias may consequently result in inappropriate health care decisions by policy makers and clinicians, which harm patients, waste resources, and misguide future research [4,5,34].

### Trial registration and public access to study data

There is an ethical obligation to publish research findings [120,147-150]. For example, patients who participate in clinical trials do so in the belief that they are contributing to medical progress, and this will only be the case if these trials are published. Deliberate non- or selective reporting represents unethical behaviour and scientific misconduct [34,147]. Public access to study data may also help identify safety problems at an earlier stage, which in the past have in some cases not always been detected by regulatory authorities [151-153]. Two concepts can help solve the issue of reporting bias: firstly, the mandatory and prospective registration of clinical trials, and secondly, the mandatory publication of full study results in results databases after study completion.

#### Non-industry initiatives

One of the first searchable computerized international registries of clinical trials was introduced in the United States in 1967; since then, several national and international trial registries have been created [154], such as the US government's trial registry and results database ClinicalTrials.gov (see Tse et al for an update on this registry [155,156]). The various controversies surrounding reporting bias, particularly the non-reporting of safety data, accelerated the movement both for trial registration and the establishment of results databases. Numerous researchers, organizations, regulatory and governmental authorities started various initiatives to achieve these goals [148,157-165].

In 2004, the International Committee of Medical Journal Editors (ICMJE) announced that it would make registration of clinical trials in a public registry a condition of consideration for publication [158]; this statement has since been updated [166,167].

In 2006, the WHO established the International Clinical Trials Registry Platform (ICTRP) in an initiative to bring national trial registries together in a global network providing a single point of access to registered trials [157]. However, to date no consensus has been found between the parties involved concerning which characteristics must be made publicly available at registration [168].

Section 801 of the US FDA Amendments Act 2007 (FDAAA, [169]) requires the registration at inception of all clinical trials involving a drug, biological product, or device regulated by the FDA. Trials must be registered on ClinicalTrials.gov and a defined set of results must be posted in the same registry within 12 months of study completion. Exceptions are phase I drug trials and early feasibility device trials. Non-compliance is sanctioned with monetary fines [163,170].

In 2004, the European Agency for the Evaluation of Medicinal Products (now European Medicines Agency) launched the European clinical trials database EudraCT (eudract.emea.europa.eu) to provide national authorities with a common set of information on clinical trials conducted in the EU. The database was initially supposed to be available only to the responsible authorities of the member states, as well as to the European Commission and the European Medicines Agency [171]. In 2006, the regulation on medicinal products for paediatric use was published, which required that information about European paediatric clinical trials of investigational medicinal products was to be made publicly available on EudraCT [172,173], and in February 2009, the European Commission published a guideline including the list of data fields to be made public [174]. On the same date, a similar list was published for all trials [175]. However, the legal obligation to publish information on trials in adults is not fully clear, and it is also unclear when all relevant information from EudraCT will be made publicly accessible.

With the introduction of the above-mentioned legislation, regulatory agencies are on the one hand helping to solve the problem of reporting bias, but on the other hand, they are also part of the problem: several of the examples identified refer to the non-publication or active withholding of study data by regulatory agencies [83,94,109,133]. This is partly due to existing confidentiality regulations such as Exemption 4 of the US Freedom of Information Act [176]. To solve the problems resulting from this situation, current legislation has to be changed to allow for the publication of comprehensive information on study methods and results by regulatory agencies. In his essay "A taxpayer-funded clinical trials registry and results database", Turner called for increased access to the FDA information sources, which would at least enable the assessment of drugs marketed in the USA [92]. Although the FDA posts selected reviews of NDAs on its website after the approval process following the Electronic Freedom of Information Act [177], the availability of these reviews is limited [92]. Moreover, according to the FDAAA, the results of older trials of approved drugs or of drugs that were never approved need not be disclosed [170], which is why a retrospective registry and results database is needed [178].

#### Industry initiatives

In 2002, the US Pharmaceutical Research and Manufacturers Association (PhRMA) member companies committed themselves to the registration of all hypothesis-testing clinical trials at initiation and to the timely disclosure of summary results, regardless of outcome [179,180]. PhRMA also launched the clinical study results database ClinicalStudyResults.org in 2004. In 2005, a similar commitment was made by several pharmaceutical industry associations [181], which has since been updated [182]. Following the legal settlement in the paroxetine case, GSK established a trial registry on its website gsk-clinicalstudyregister.com and other large companies have followed. In 2008, the German Association of Research-Based Pharmaceutical Companies (VFA) published a position paper on the issue of publication bias and claimed that, because of the voluntary self-commitment of the pharmaceutical industry and the introduction of legislation for the reporting of study data, publication bias had become a "historical" topic [183]. However, even after the update of the position paper in January 2009 [184], in Germany alone further attempts by drug companies to withhold study data have occurred [69], which shows that voluntary self-commitment is insufficient.

## Conclusions

Reporting bias is widespread in the medical literature and has harmed patients in the past. Mandatory prospective registration of trials and public access to study data via results databases need to be introduced on a worldwide level. This would help fulfil ethical obligations towards patients by enabling proactive publication and independent reviews of clinical trial data, and ensure a basis for fully informed decision making in the health care system. Otherwise, clinical decision making based on the "best evidence" will remain an illusion.

## Competing interests

Non-financial competing interests: All authors are employees of the German Institute for Quality and Efficiency in Health Care. In order to produce unbiased HTA reports, the Institute depends on access to all of the relevant data on the topic under investigation. We therefore support the mandatory worldwide establishment of trial registries and study results databases.

## Authors' contributions

NM and BW had the idea for the manuscript. NM, HK, YBS, and JK screened reference lists. JK and YBS reviewed titles and abstracts of potentially relevant citations identified in the screening process. NM extracted relevant examples from the full-text publications. BW and TK checked the extracted examples. NM drafted the first version of the manuscript. The remaining authors contributed important intellectual content to the final version. All authors approved the final version.

## Supplementary Material

Additional file 1 **Table S2: Examples of reporting bias in the medical literature**. Extracts from 50 publications presenting examples of reporting bias. Click here for file
