# Supplementary material for: Reporting bias in medical research - a narrative review
Source: Trials. 2010 Apr 13;11:37. doi: 10.1186/1745-6215-11-37 (PMC2867979; doi:10.1186/1745-6215-11-37)
Supplement: Additional file 1 — Table S2: Examples of reporting bias in the medical literature. Extracts from 50 publications presenting examples of reporting bias. [file 1745-6215-11-37-S1.DOC]

**Table S2: Examples of reporting bias in the medical literature**

| **Indication** | **Intervention** | **Document type, citation** | **Main findings** | **Further reading** |
| --- | --- | --- | --- | --- |
| **Mental and behavioural** |  |  |  |  |
| Depression | Antidepressants | Journal article comparing reviews from the FDA and matching publications [16] | “Among 74 FDA-registered studies, 31%, accounting for 3449 study participants, were not published…. A total of 37 studies viewed by the FDA as having positive results were published…. Studies viewed by the FDA as having negative or questionable results were, with 3 exceptions, either not published (22 studies) or published in a way that, in our opinion, conveyed a positive outcome (11 studies). According to the published literature, it appeared that 94% of the trials conducted were positive. By contrast, the FDA1 analysis showed that 51% were positive. …the increase in effect size ranged from 11 to 69% for individual drugs and was 32% overall” [16]. | [14, 94, 185] |
|  |  | Journal article on a meta-analysis of data submitted to the FDA [83] | “Although sponsors are required to submit information on all trials, the FDA public disclosure did not include mean changes for nine trials that were deemed adequate and well controlled but that failed to achieve a statistically significant benefit for drug over placebo. Data for four of these trials were available from a pharmaceutical company Web site in January 2007 and were obtained from the GlaxoSmithKline clinical trial register… Specifically, four sertraline trials involving 486 participants and one citalopram trial involving 274 participants were reported as having failed to achieve a statistically significant drug effect, without reporting mean HRSD scores. We were unable to find data from these trials on pharmaceutical company Web sites or through our search of the published literature. These omissions represent 38% of patients in sertraline trials and 23% of patients in citalopram trials” [83]. |  |
|  | - SSRIs | Journal article: systematic review of published vs. unpublished data [70] | “Data for two published trials suggest that fluoxetine has a favourable risk-benefit profile, and unpublished data lend support to this finding. Published results from one trial of paroxetine and two trials of sertraline suggest equivocal or weak positive risk-benefit profiles. However, in both cases, addition of unpublished data indicates that risks outweigh benefits. Data from unpublished trials of citalopram and venlafaxine show unfavourable risk-benefit profiles” [70]. | [151, 186-189] |
|  | -- Paroxetine | Press release on the concealment of study data [84] | “..GSK conducted at least five studies on the use of Paxil [paroxetine] in children and adolescents. However, GSK only published and disseminated one of these studies, which showed mixed results on efficacy. The lawsuit alleges that the company suppressed the negative results of the other studies, which failed to demonstrate that Paxil is effective and which suggested a possible increased risk of suicidal thinking and acts” [84]. | [85, 86, 190, 191] |
|  | - Reboxetine | Press release on the concealment of study data [192] | “…reboxetine …was tested in at least 16 trials on approximately 4600 patients with depression. However, the Institute only had access to data on approximately 1600 of these patients” [192] | [69, 87] |
|  | - Mirtazapine | See above | “…it was found that this agent had been tested in at least 31 trials that could be potentially relevant to the report. However, the Institute only had access to 27 evaluable trials” [192]. | [69, 87] |

*cont’d*

**Table S2 (cont’d): Examples of reporting bias in the medical literature**

| **Indication** | **Intervention** | **Document type, citation** | **Main findings** | **Further reading** |
| --- | --- | --- | --- | --- |
| Bipolar disorder | Lamotrigine | Journal article comparing commercial / public registries and publications [35] | “Two negative studies have been published, 1 in rapid-cycling [ref] and another in acute bipolar depression [ref], but both published versions emphasize positive secondary outcomes as opposed to the negative primary outcomes. A negative study in rapid cycling has not been published in detail (GW611), nor have 2 negative randomized studies in acute bipolar depression (GW 40910 and GW 603), or 2 negative randomized trials in acute mania (GW 609 and GW 610). However, all of these negative studies are reported at the company Web site, which also refers to a publication [ref] that briefly summarizes results from the above 5 negative studies.” “…the drug has very limited, if any, efficacy in acute bipolar depression and rapid-cycling bipolar disorder…” [35]. |  |
|  | Gabapentin | Expert report on reporting and other biases [34] | “Two of the three studies of Neurontin [gabapentin] for bipolar disorders had ‘negative’ results for the primary outcome, and all three were published. The study with ‘positive’ results used an open label design which is not useful for determining efficacy. The bipolar publications were marked by extensive spin and misrepresentation of data” [34]. | [89, 90, 193-198] |
| Schizophrenia | Quetiapine | Newspaper report on the suppression of study results [91] | “The results of Study 15 were never published or shared with doctors…internal documents show that company officials were worried because 45 percent of the Seroquel [quetiapine] patients had experienced what AstraZeneca physician Lisa Arvanitis termed "clinically significant" weight gain…In an e-mail dated Aug. 13, 1997, Arvanitis reported that across all patient groups and treatment regimens, regardless of how numbers were crunched, patients taking Seroquel gained weight: ‘I'm not sure there is yet any type of competitive opportunity no matter how weak.’ In a separate note, company strategist Richard Lawrence praised AstraZeneca's efforts to put a "positive spin" on "this cursed study" and said of Arvanitis: ‘Lisa has done a great 'smoke and mirrors' job!’ Two years after those exchanges, in 1999, the documents show that the company presented different data at an American Psychiatric Association conference and at a European meeting. The conclusion: Seroquel helped psychotic patients lose weight” [91].. | [199-201] |
| Generalized anxiety disorder | Paroxetine | Journal article on the call for more freedom of information at the FDA [92] | “A Cochrane systematic review of antidepressants for generalized anxiety disorder [ref] lists only one double-blind placebo-controlled study of paroxetine [ref], a positive study. A PubMed search reveals no additional double-blind placebo-controlled studies. In accessing the review from Drugs@FDA (approval date April 2001), we learn that there were in fact three pivotal double-blind placebo-controlled studies. One of these studies corresponds to the published positive study noted above. Of the remaining two studies, both apparently unpublished, one was positive while the other was marginally positive” [92]. | [202] |
| Panic disorder | Paroxetine | See above | “Turning to the controlled-release formulation of paroxetine (Paxil CR) for panic disorder, a review article states in its abstract that the drug “demonstrated efficacy in three well designed studies in patients with panic disorder with or without agoraphobia” [ref]. In reading the corresponding FDA statistical review, we verify that there were indeed three studies. However, the FDA statistical reviewer found that only one of these studies was strongly positive. A second study, judged ‘supportive’ of efficacy, had a marginally significant (p = 0.039) result on a secondary observed-cases analysis, but a nonsignificant (p = 0.38) result on the primary efficacy analysis defined a priori. The third study was clearly negative, with p-values of 0.33 and 0.57 on the primary and secondary analyses, respectively” [92]. | [203] |

*cont’d*

**Table S2 (cont’d): Examples of reporting bias in the medical literature**

| **Indication** | **Intervention** | **Document type, citation** | **Main findings** | **Further reading** |
| --- | --- | --- | --- | --- |
| Attention-deficit hyperactivity disorder | ADHD drugs | Online article on government-funded data concealment [204] | “In another NIH study, published in 2003, researchers reported that the brains of children with attention-deficit hyperactivity disorder (ADHD) were smaller than those of children who had not been diagnosed with the disorder. Critics questioned whether the problem might actually be caused by the drugs used to treat ADHD. But, when a researcher asked for the underlying data, his request was denied.……In the case of the study showing that the brains of children with ADHD are smaller, it means that stimulant drugs continue to be prescribed even though it is not clear whether brain shrinkage in children with ADHD is caused by the disease--or by the drugs used to treat the disease” [204] |  |
| **Nervous system** |  |  |  |  |
| Alzheimer’s disease | Rofecoxib | Journal article on a case study based on litigation documents [74] | “….the company's internal intention-to-treat analyses of pooled data from these 2 trials identified a significant increase in total mortality (hazard ratio [HR], 4.43; 95% CI, 1.26-15.53 for protocol 091, and HR, 2.55; 95% CI, 1.17-5.56 for protocol 078), with overall mortality of 34 deaths among 1069 rofecoxib patients and 12 deaths among 1078 placebo patients (HR, 2.99; 95% CI, 1.55-5.77). These mortality analyses were neither provided to the FDA nor made public in a timely fashion” [74]. | [73, 105-107, 152, 205-215] |
| Pain (acute) | Valdecoxib | Journal article on the withholding of study data [94] | “Take valdecoxib, a COX 2 inhibitor that was withdrawn from the market because it posed a serious risk of heart attacks. [ref] In 2004 the health research group of Public Citizen, a non-profit, public interest organisation in Washington, DC, tried to assess the drug’s safety profile. In 2001 the manufacturer applied for approval to market valdecoxib for four indications: osteoarthritis, dysmenorrhoea, adult rheumatoid arthritis, and acute pain. The FDA approved the drug for the first three indications but not for acute pain, and some of the information about the acute pain trials was withdrawn from the FDA website and a statement given that the information contained ‘trade secret and/or confidential information that is not disclosable’” [94]. | [109] |
| Pain (neuropathic) | Gabapentin | Expert report on reporting and other biases [34] | “Four of the nine randomized trials conducted for treatment of neuropathic pain had negative findings, and seven were published, four with ’positive’ and three with ’negative’ results. As with all the trials I reviewed, selective analyses (i.e., no intention to treat analyses, despite the company’s saying so) could explain any positive findings observed… Published studies with negative findings were presented with considerable ‘spin’ and misrepresentation of data” [34]. | [89, 90, 193-198] |
| Pain (nociceptive) | Gabapentin | See above | “None of the five+ studies of Neurontin [gabapentin] combined with other analgesics for nociceptive pain showed a statistically significant benefit of Neurontin when it was added to either naproxen or hydrocodone. And none of these studies, all with negative results, were published. When a statistically significant benefit of a Neurontin combination regimen *was* found to be beneficial compared to placebo, it was apparent that the beneficial effect was due to the naproxen or hydrocodone not the Neurontin” [34]. |
| Migraine | Gabapentin | See above | “Three studies were conducted but the final results were only published for one [ref]. The final results for all three studies were negative. Nevertheless, positive preliminary results were published for one study, and one study was published in full, after a long time lag. In this study (945-220), statistically significant primary results were presented in the article and this was not consistent with the findings in the research report” [34]. |

*cont’d*

**Table S2 (cont’d): Examples of reporting bias in the medical literature**

| **Indication** | **Intervention** | **Document type, citation** | **Main findings** | **Further reading** |
| --- | --- | --- | --- | --- |
| **Circulatory system** |  |  |  |  |
| Myocardial infarction | Psychological rehabilitation | HTA report on publication and related biases [38] | “In a meta-analysis of psychological rehabilitation after myocardial infarction, [ref] mortality was significantly lower in the rehabilitation group than in the usual care group (RR 0.65; 95% CI 0.46, to 0.91), according to the results of eight trials that reported total mortality. By contacting the principal investigators of three other trials that did not report total mortality, data were received and the revised RR became 0.73 with a less significant CI (95% CI 0.53 to 1.00). No difference in mortality was observed in a subsequent large multicentre trial (RR 1.01; 95% CI 0.75 to 1.37)” [38]. | [216] |
| Cardio-vascular disease (bleeding prophylaxis during bypass surgery) | Aprotinin | Journal article on the absence of transparency in observational studies on drug safety [96] | “However, in early 2006, two observational studies were published that raised serious concerns about the drug’s safety…Unfortunately, Mangano did not give the FDA or the committee full access to his data, which would have allowed the agency to perform an independent review and analysis to validate his group’s findings….. Days after the committee meeting, the FDA was made aware of additional observational data from the sponsor that had not been presented at the meeting. Bayer evidently had commissioned an observational study involving 67,000 patients who were given aprotinin. [ref] According to the initial FDA review of data from that study, aprotinin may be associated with ‘increased risk for death, kidney failure, congestive heart failure and stroke’. The failure of Bayer to disclose all its data on aprotinin seriously undermined the advisory committee process and hindered the safety review” [96]. | [97, 217, 218] |
| Prevention of arrhythmia | Class I anti-arrhythmic drugs | Book on class I anti-arrhythmic drugs [72]; study publication [71] | The book “Deadly medicine” [72]: “America’s worst drug disaster” …“produced a death toll larger than the United States’ combat losses in wars such as Korea and Vietnam”. Primary study: “…there were nine deaths among the 49 patients treated with lorcainide compared with only one in the patients given placebo. ….This study was carried out in 1980 but was not published at the time: it now provides an interesting example of 'publication bias'” [71]. | [98-101] |
| Prevention of sudden cardiac death | Implantable cardioverter-defibrillator | Journal article on the withholding of safety information [219] | “At the time of our patient’s death, Guidant [the manufacturer] had knowledge of 25 similar Prizm 2 DR model 1861 failures in patients, 3 of whom had required rescue defibrillation. Indeed, Guidant had first observed this mode of failure 3 years earlier, in February 2002, when 2 returned Prizm 2 DR pulse generators exhibited the same short circuiting that caused our patient’s device to fail. Guidant was sufficiently concerned about these failures that manufacturing changes were made in April and November of 2002, which allegedly prevented short circuiting. Nevertheless, Guidant chose not to inform patients or physicians about these failures or the manufacturing changes designed to prevent them. Moreover, Guidant continued to sell pulse generators that were built before the 2002 manufacturing changes. Unknowingly, therefore, we and other physicians implanted Prizm 2 DR ICDs in 2002 and 2003 that Guidant knew were prone to sudden unexpected failure” [219]. |  |
| Congestive heart failure | Nesiritide | Journal article on secrecy in medical research [220] | “In 2001 the FDA approved nesiritide (Natrecor) for treatment of congestive heart failure, despite data hinting that the drug was associated with impaired renal function and increased mortality relative to other intravenous therapies. [ref] Although the FDA requested that the manufacturer, Scios, further study the incidence of such side effects, it did not delay nesiritide's approval. Four years later, researchers not affiliated with the FDA used meta-analyses of the summaries of initial data and data published after its approval to show that the side effects and mortality rates were statistically significant and clinically troubling…. a more comprehensive analysis could have been conducted had the analysts had access to the full set of data submitted to the FDA” [220] | [221] |

*cont’d*

**Table S2 (cont’d): Examples of reporting bias in the medical literature**

| **Indication** | **Intervention** | **Document type, citation** | **Main findings** | **Further reading** |
| --- | --- | --- | --- | --- |
| Ischaemic brain infarction | Unknown product | Journal article on bias in reporting clinical trials [17] | “Treatment of ischaemic brain infarction with one product resulted in increased mortality; this outcome was neither reported to the Finnish authority nor published in any journal “ [17]. |  |
| **Digestive system** |  |  |  |  |
| Gastric ulcer | Prostaglandin analogues | Letter to the editor on the suppression of study results [64] | “A double-blind, randomised, multicentre, multinational trial design was developed in December, 1983, by a drug company to compare their investigational synthetic prostaglandin analogue with ranitidine in patients with endoscopically proven gastric ulcer. …In December, 1984, a Danish centre agreed to join the trial and recruited 13 patients between April, 1985, and the end of August, 1985, when trial was stopped because the stipulated number of patients had been included. In March, 1986, the Danish investigators asked for the trial report and were informed by local company officials that …preliminary analyses had demonstrated that the investigational drug produced significantly lower healing rates than ranitidine …In April, 1987, the Danish investigators had still not received the complete report” [64] |  |
| Irritable bowel syndrome | Alosetron | Letter to the editor on selective reporting [103] | “The graphic techniques used by Camilleri and colleagues greatly exaggerate alosetron's efficacy. Their figure 3 presents the relative difference in pain and discomfort scores from baseline on a 0—4 scale for the treated and placebo groups. Presented this way, the drug seems effective. However, when we plotted the absolute data contained in the Medical Officer's Review, the data points are almost superimposable ([figure](http://www.thelancet.com/journals/lancet/article/PIIS0140-6736(05)72978-0/fulltext#fig1%23fig1)). The exclusion of the baseline data from Camilleri and colleagues' figure 3, unusual in a graph of this type, has the additional effect of enlarging the Y axis, and creating an apparent greater benefit for the drug” [103]. | [104, 222, 223] |
| **Genito-urinary system / perinatal** |  |  |  |  |
| Stress urinary incontinence | Duloxetine | Journal article on the failure to disclose study data [94] | “This aspect of drug regulation surfaced in 2005, with the death of 19 year old Traci Johnson, who committed suicide while serving as a healthy volunteer in a trial of duloxetine for a new indication, urinary incontinence. After requesting the data on duloxetine from the FDA, one of us (Lenzer) found that Johnson’s death, in addition to those of at least four other volunteers, was not included. When questioned, the FDA cited trade secrecy laws, [ref] which permit companies to withhold all information, even deaths, about drugs that do not win approval for a new indication, even when the drug is already on the market for other indications” [94]. | [170, 224, 225] |
| Pregnancy | Preventive intervention  (bed rest in uncom-plicated twin pregnancy) | Journal article on underreporting of studies [147] | “The first randomized evaluation of hospitalization for bed rest in uncomplicated twin pregnancy was conducted in Harare, Zimbabwe, in 1977. ....A preliminary analysis of the trial suggested that, far from reducing the risk of preterm delivery, routine hospitalization was actually associated with an increased rate of this outcome. ….The results of the trial would have remained unreported if it had not been for the fact that, 7 years later, two visitors to Harare “discovered” these unpublished data and helped the investigators to analyze and report their unreported trial. ….The results of this trial, taken together with comparable findings in a similar trial….provoked reevaluation of an obstetric policy that has been widely accepted for four decades”…..” [147] | [226] |

*cont’d*

**Table S2 (cont’d): Examples of reporting bias in the medical literature**

| **Indication** | **Intervention** | **Document type, citation** | **Main findings** | **Further reading** |
| --- | --- | --- | --- | --- |
|  | Diagnostic intervention (routine ultrasound) | see above | “..The confusion in this field is compounded by the fact that one large trial of routine ultrasonography, conducted nearly 10 years ago, remains unpublished. ….Assessments of the effects of routine ultrasonography in pregnancy is also bedeviled by inadequate published reports of relevant trials. The only randomised trial to suggest that routine ultrasongraphy has any beneficial effects on substantive outcomes of pregnancy …..has never been fully reported in a scientific journal. …The reports that are available are contradictory in a very important aspect….This inconsistency between the only two reports of this trail is clearly of great importance in any attempt to answer the still inadequately addressed question of whether routine ultrasonography is preferable to selective ultrasonography….” [147]. |  |
| **Musculo-skeletal system** |  |  |  |  |
| Osteoarthritis | Rofecoxib | Journal article: review of FDA data and study publications [107] | Results of the VIGOR trial (according to FDA reports): Frequency of serious cardiovascular thrombotic events in patients treated with naproxen vs rofecoxib: 0.7% vs. 1.7* (p < 0.05) [107]. These data were not reported in the study publication, which reported the following: “The incidence of myocardial infarction was lower among patients in the naproxen group than among those in the rofecoxib group (0.1 percent vs. 0.4 percent; relative risk, 0.2; 95 percent confidence interval, 0.1 to 0.7)…The finding that naproxen therapy was associated with a lower rate of myocardial infarction needs further confirmation in larger studies” [105]. | [73, 74, 106, 152, 205-215] |
|  | Celecoxib | Journal article on access to pharmaceutical data at the FDA [109] | “In another revealing example, the Journal of the American Medical Association published a report in 2001 claiming that, after six months of therapy, the COX-2 inhibitor celecoxib (Celebrex) was associated with a reduced incidence of gastrointestinal ulcers compared to two other pain medications. [ref] If true, this outcome would have represented a significant advantage over other approved pain medications. However, the authors of the study failed to disclose that at the time of publication they had already received data for the full twelve-month period for which the study was originally designed. [ref] The twelve-month data showed no advantage for Celebrex over other drugs. Although the FDA, armed with the twelve-month data, has never allowed the company to claim reduced ulcer incidence, the published study helped drive the massive Celebrex market” [109]. | [108] |
| Skin |  |  |  |  |
| Atopic dermatitis | Evening primrose oil | Journal article (commentary) on the suppression of study results [66] | “…in 1995 the Department of Health commissioned me and a colleague to conduct an individual patient meta-analysis of 20 studies of oral evening primrose oil supplementation for treatment of atopic dermatitis, which included 10 unpublished studies held by the company…. Although it was our view that the report produced a relatively clear conclusion, we were never allowed to share the report in the public domain…. Shortly after we submitted our report to the Department of Health, Searle, the company then responsible for marketing evening primrose oil, expressed concern that the contents of the report had been leaked, and the authors and referees were required to sign a written statement to the company (through the Department of Health) to indicate that this was not the case” [66]. |  |

*cont’d*

**Table S**2 (cont’d): Examples of reporting bias in the medical literature

| **Indication** | **Intervention** | **Document type, citation** | **Main findings** | **Further reading** |
| --- | --- | --- | --- | --- |
| **Endocrine / metabolic system** |  |  |  |  |
| Diabetes mellitus type 2 | Rosiglitazone | Online article (conversation about rosiglitazone between 2 US physicians) [110] | Dr. Nissen: “GlaxoSmithKline …had taken 42 clinical trials …and had performed their own integrated analysis. It reported to the FDA in August of 2005 that their analysis suggested a 31% increase in the risk of ischemic cardiovascular complications. …the company did in fact post on a Web site the results of their meta-analysis showing a 31% increase in risk. We do know from FDA documents that the company told the FDA in August of 2005, and again a year later, that they were seeing very strong evidence in their own meta-analysis of an increased risk of myocardial infarction and other ischemic events. But for whatever reasons, neither the company nor the FDA revealed that in a public way” [110]. | [111, 227-237] |
|  | Metformin | Journal article on access to pharmaceutical data at the FDA [109] | “…Public Citizen sought the protocol for a Phase IV study of metformin (Glucophage®). The FDA had required the study to establish the incidence of a drug-induced metabolic disorder called lactic acidosis. The protocol would provide a detailed description of the study design, including its inclusion criteria, length of follow-up, outcome measures and statistical procedures. Bristol-Myers Squibb, the drug’s manufacturer, intervened in the lawsuit. Both Bristol-Myers and FDA argued that the protocols were properly withheld under the confidential commercial information FOIA exemption.....The court sought the input of two independent experts, and both agreed that release of the protocol would not be likely to harm the company at all. The court then ordered the agency to release the protocol” [109]. |  |
|  | Muraglitazar | Journal article on secrecy in medical research [220] | “Another example occurred in September 2005 when an FDA advisory committee recommended approval of muraglitazar (Pargluva) for the treatment of diabetes and dyslipidemia. Soon after, other researchers reanalyzed the summary data released by the FDA and found statistically significant evidence of increased risk of cardiovascular complications and mortality. [ref] Bristol-Myers Squibb removed the drug from FDA consideration ... a more comprehensive analysis could have been conducted had the analysts had access to the full set of data submitted to the FDA” [220]. |  |
| Hypercholest-erolaemia | Ezetimibe and simvastatin | Journal article reporting a publication delay [112] | “The trial pitted Vytorin, a combination of ezetimibe … and simvastatin … against simvastatin alone in patients with heterozygous familial hypercholesterolemia. After 2 years, the investigators found no statistically significant difference between treatment groups on the primary end point of mean change in the carotid intima media thickness…. These findings …came amid growing controversy about the nearly 2-year delay in reporting the results after the trial’s conclusion in April 2006” [112]. | [113, 238-241] |
|  | Ezetimibe | Newspaper article reporting the withholding of study results [241] | “New evidence shows that the drug makers [Merck](http://topics.nytimes.com/top/news/business/companies/merck_and_company/index.html?inline=nyt-org) and [Schering-Plough](http://topics.nytimes.com/top/news/business/companies/schering_plough_corporation/index.html?inline=nyt-org) have conducted several studies of their popular [cholesterol](http://health.nytimes.com/health/guides/nutrition/cholesterol/overview.html?inline=nyt-classifier) medicine Zetia that raise questions about its risks to the liver, but the companies have never published those results. … The unpublished studies, conducted from 2000 to 2003 according to the F.D.A. documents, were not listed on the industry Web sites where companies are supposed to register the results of all drug trials that were ongoing after October 2002. The New York Times discovered references to the studies in briefing papers on the F.D.A. Web site” [241]. | [112, 113, 238-240] |

*cont’d*

**Table S**2 (cont’d): Examples of reporting bias in the medical literature

| **Indication** | **Intervention** | **Document type, citation** | **Main findings** | **Further reading** |
| --- | --- | --- | --- | --- |
|  | Cerivastatin | Journal article: review of internal company documents and published literature [114] | “In internal company documents, multiple case reports suggested a drug-drug interaction within approximately 100 days of the launch in 1998; however, the company did not add a contraindication about the concomitant use of cerivastatin and gemfibrozil to the package insert for more than 18 months. Unpublished data available in July 1999 also suggested an increased risk of rhabdomyolysis associated with high doses of cerivastatin monotherapy. In late 1999 and early 2000, company scientists conducted high-quality analyses of the US Food and Drug Administration adverse event reporting system data. These analyses suggested that compared with atorvastatin calcium, cerivastatin monotherapy substantially increased the risk of rhabdomyolysis. To our knowledge, these findings were not disseminated or published” [114]. | [115, 242-245] |
| Thyroid  disorders | Levothyroxine | Journal article (news report) on the suppression of study results [65] | “In 1987 Betty Dong and colleagues …were asked by Flint Laboratories, the manufacturer of Synthroid, to carry out research comparing their drug with three others…. By the end of 1990, when the study was complete and it became clear that all four preparations were bioequivalent, the results were sent off to Boots Pharmaceuticals, which had taken over Flint Laboratories. Dr Rennie says that over the next four years Boots ‘waged an energetic campaign to discredit the study and prevent its publication.’ The study was eventually submitted to JAMA in April 1994…. On 13 January 1995 Dr Dong suddenly withdrew her manuscript from publication, citing impending legal action by Boots... Under pressure from the FDA Knoll agreed on 25 November 1996 to allow the research to be published…” [65]. | [116, 117, 246] |
| Menopausal  symptoms | Tibolone | Online article of a TV report on the publication delay of study results [119] | Translation from German: “The study was only made public 1.5 years later. According to the manufacturer Essex-Organon in a comment to Frontal 21 (*German TV programme*), this corresponds to ‘good scientific practice’ and anyway, the disclosure had been made ‘according to the valid rules’ and ‘according to regulations’. Critics are not satisfied with this: ‘I think it is irresponsible that a company does not disclose negative results immediately, as in the case of breast cancer caused by a product, and also does not immediately inform physicians’ said Becker-Brüser (*publisher of the German Drug Telegram*)” [119]. Primary study: “Tibolone increases the risk of recurrence in breast cancer patients….” [118]. |  |
| Neoplasms |  |  |  | [40, 50, 120, 122-125] |
| Ovariancancer | Combination chemotherapy | Journal article: analysis of registry studies vs published studies [121] | Survival impact of initial alkylating agent (AA) vs combination chemotherapy (CC): “…a pooled analysis of published clinical trials demonstrates a significant survival advantage for combination chemotherapy (median survival ratio of CC to AA, 1.16; P = .02). However, no significant difference in survival is demonstrated based on a pooled analysis of registered trials (median survival ratio, 1.05; P = .25)” [121]. |  |
| Multiplemyeloma | Combination chemotherapy | Journal article (see above) [121] | Survival impact of AA/prednisone vs CC: ..”a pooled analysis of published trials also demonstrates a significant survival advantage for CC (median survival ratio, 1.26; P = 04), especially for poor risk patients (ratio, 1.66; P = .002). A pooled analysis of registered trials also shows a survival benefit for patients receiving combination chemotherapy (all patients, P = .06; poor risk, P = .03), but the estimated magnitude of the benefit is reduced (all patients: ratio, 1.11; poor risk: ratio, 1.22)” [121]. |  |

*cont’d*

### Table S2 (cont’d): Examples of reporting bias in the medical literature

| **Indication** | **Intervention** | **Document type, citation** | **Main findings** | **Further reading** |
| --- | --- | --- | --- | --- |
| Melanoma | Drug therapy | Journal article on publication bias and dissemination of clinical research [247] | “Another study (Dubrow M, Begg CB: unpublished data) involved phase II trials of treatments for melanoma. The design was similar to that of the Simes study [ref], even though the trials were not comparative. The same sources were used: MEDLINE searches of published articles and the NCI registry of cancer trials. Published and registered trials were matched on the basis of the drugs used in the study, the institution or cooperative group that conducted the study, and the sequence of dates of registration and publication. A total of 141 registered trials and 241 published trials were identified ……… The response rates in the nonregistered studies are clearly higher in all categories. However, the most striking result of this study is the observation that only 46 (33%) of the 141 registered trials were published.” Response rate (sample size > 40); 13% vs, 19% /registered vs. non-registered) [247]. (Comment: larger differences were noted between trials with small sample sizes; these results are not considered here). |  |
| Blood system |  |  |  |  |
| Thalassaemia major | Iron chelation | Journal article (editorial) on the attempted suppression of study results [128] | “In 1995, Dr Nancy Olivieri published an optimistic article on the effects of an oral iron-chelation agent. [ref, ref] As her trials proceeded, however, she became disturbed by the agent’s lack of effectiveness……and she was concerned about possible danger to patients. ….She decided she had to break confidentiality by reporting her results at a meeting. [ref, ref] The manufacturers disagreed with her interpretation of the results and tried unsuccessfully to block her presentation” [128]. | [248] |
| **Bacterial, fungal, and viral infections** |  |  |  |  |
| Bacterial | Telithromycin | Newspaper report on safety concerns surrounding the approval of an antibiotic [249] | “The company first sought approval for Ketek [telithromycin] in February 2000. But hints in the company’s clinical trials that it could cause liver problems led the agency to ask for more information. The company began a study in 24,000 patients, but F.D.A. investigators found repeated instances of fraud in the study. One agency document concluded that Sanofi-Aventis knew of some of the problems in this trial but failed to alert the F.D.A. before the agency discovered the problems on its own. The document also concluded that the company’s required reports about problems with the drug ‘have been incomplete or reported in a dilatory fashion’” [249]. | [250] |
| Fungal | Fluconazole | Journal article on reporting of trials of antifungal agents [251] | “In 3 large trials that comprised 43% of the patients identified for the meta-analysis, results for amphotericin B were combined with results for nystatin in a “polyene” group. Because nystatin is recognized as an ineffective drug in these circumstances, this approach creates a bias in favor of fluconazole.... It was unclear whether there was overlap among the “polyene” trials, and it is possible that results from single-center trials were included in multicenter trial reports. We were unable to obtain information to clarify these issues from the trial authors or the manufacturer of fluconazole. Two of 11 responding authors replied that the data were with the drug manufacturer and two indicated that they did not have access to their data because of change of affiliation” [251]. |  |
| Viral |  |  |  |  |

*cont’d*

**Table S**2 (cont’d): Examples of reporting bias in the medical literature

| Indication | **Intervention** | **Document type, citation** | **Main findings** | **Further reading** |
| --- | --- | --- | --- | --- |
| *Influenza* | Oseltamivir | Editorial on difficulties in obtaining data for a Cochrane review [129] | “Jefferson et al noted that the Kaiser analysis was funded by the drug’s manufacturer Roche and was based entirely on 10 trials funded by Roche, only two of which had been published as articles in peer reviewed journals…Jefferson et al asked for clarification from the authors, who directed them to Roche. After initially declining to provide the necessary data, staff at Roche sent the Cochrane reviewers a selection of files, which answered some questions but still left the reviewers unable to reconstruct the Kaiser dataset. Of particular concern, the eight unpublished trials, involving 2691 patients, included one of the biggest trials of oseltamivir. More worrying still, the academic author named on the trial’s abstract in the Kaiser analysis and in company documents has told the BMJ he was not involved in the trial” [129]. | [130-132] |
| *Hepatitis B* | Fialuridine | Journal article on access to pharmaceutical data at the FDA [109] | “…Public Citizen sought records regarding preclinical and clinical testing of fialuridine, a drug that had caused the deaths of five patients in clinical trials for Hepatitis B infection at the National Institutes of Health. [ref] Despite this striking toxicity, the drug’s sponsor, Eli Lilly, claimed that it would continue to develop the drug. [Footnote: The drug had never been approved for use in any condition.]. The FDA and the drug’s sponsor argued that the materials sought were subject to the confidential commercial information exemption. The court agreed, noting in an unreported decision that the records fell within the scope of that exemption because the information they contained might save Lilly’s competitors both time and money in doing their own research” [109]. | [252] |
| *HIV/AIDS* | Antivirals | Journal article on the reporting of HIV trials [33] |  | [18] |
|  | - Zidovudine | see above | “ACTGOOl, a trial of 273 patients on zidovudine versus placebo in patients with Kaposi’s sarcoma, remains unpublished since completion in 1991” [33]. |  |
|  | - Zalcitabine | see above | “ACTG114, a negative trial of 668 patients comparing zalcitabine with zidovudine, remains unpublished since its meeting presentation in 1993; data on a quality of life substudy have been published” [33]. |  |
|  | - Ribavirin | see above | “A Canadian ribavirin trial with 469 patients (the largest trial for this medication) was prematurely interrupted at an early phase in 1990 because, in the meanwhile, zidovudine was shown to be effective” [33]. |  |
|  | - Interferon | see above | “Five trials reported at meetings in 1992 and 1993 and enrolling 112, 150, 180, 243, and 560 patients, respectively, remain unpublished as well as several smaller studies. None showed clinical efficacy in their preliminary results….two more inconclusive interferon trials with 402 and 45 patients… were brought to our attention after our paper had been accepted. Their publication was delayed for about 3 years after their completion” [33]. |  |
|  | - Acyclovir | see above | “A trial of 677 patients remains unpublished since its meeting presentation in 1992. The trial showed no efficacy” [33]. |  |
|  | - Isoprinosine | see above | “Two large trials have been unpublished. Their preliminary results showed no efficacy” [33]. |  |
| *Herpes simplex* | Famciclovir | Report article on authorship, ghost-science, access to data [253] | “A recent example involves the drug Famciclovir, used to treat herpes. A 1997 study comparing Famciclovir with its main competitor was funded by the manufacturer (then Smithkline Beecham). Study findings were not beneficial to the sponsor. They were published only a few months ago after a nine-year delay, with a disclaimer that the authors were denied raw data and were forced to accept the company’s own partial summary of findings (ref)” [253]. |  |

*cont’d*

**Table S2:** (cont’d): Examples of reporting bias in the medical literature

| **Indication** | **Intervention** | **Document type, citation** | **Main findings** | **Further reading** |
| --- | --- | --- | --- | --- |
| Acute trauma |  |  |  |  |
| Acute spinal cord injury | High-dose steroids | Journal article on the withholding of study data [94] | “Fred Geisler, a neurosurgeon at the Illinois Neuro-Spine Center, points to the use of high dose steroids in patients with spinal injury on the basis of a single, potentially flawed study funded by the National Institutes of Health (NIH). [ref] Geisler believes that several thousand patients have died as the result of high dose steroids used to treat acute spinal cord injury. Two recent surveys show that most neurosurgeons share his concerns. [ref] ….Several researchers have lobbied unsuccessfully for the release of the underlying data, without which they cannot verify their concerns—or lay them to rest” [94] | [204, 254] |
| Shock | Human albumin | Report by the UK Health Committee on industry influence [133] | Iain Chalmers: “Human albumin solution has been used repeatedly in this country, and it was re-licensed in 1993. Yet a systematic review of all of the studies that provided information about death done in the mid 90s showed no evidence that albumin was helpful, and some worrying evidence that it might be harmful. …The reaction of the Medicines Control Agency to this news was to slightly modify the labelling, but to keep confidential the evidence upon which the drug had been re-licensed in 1993” [133] . | [134] |
| Vaccinations |  |  |  |  |
|  | HIV-1 vaccine | Journal article: news report on the attempted blocking of a publication [135] | “Having failed to block publication of a HIV-1 vaccine study …a US company has now filed a claim against the study’s lead investigators and their universities asking for US$7–10 million in damages. … The trial began in the mid-1990s, but was ended early when in May, 1999, the data safety monitoring board concluded that the study was finding no difference in efficacy between the vaccine and placebo…. Immune Response, however, wanted the researchers to include in their report of the study’s findings an analysis of viral load data from a subset of patients ….But the lead investigators refused, arguing that it would be improper to include a post-hoc analysis that had not been part of the study’s original protocol and that the company had not used valid statistical methods. ….The company then filed a claim seeking to prevent publication of the study…” [135]. |  |
|  | Cancer vaccine | Journal article on secrecy in medical research [75] | “In recent studies in animals, my colleagues and I uncovered what appeared to be a promising use for a new reagent as a component of a cancer vaccine we were developing for clinical trials. The company that developed the reagent had just completed studies to determine a safe dose in humans and agreed to supply the reagent to us for our trial. The company refused to reveal the dosage information, however, and would not provide data on the toxic effects in patients with cancer in their trial unless we agreed to keep the information confidential so that a competing company would not gain access to it….researchers were asked to keep information confidential that might prevent patients with cancer from receiving ineffective or even harmful doses of a new agent” [75]. |  |
| Others |  |  |  |  |
| Nocturnal leg cramp | Quinine | Journal article: meta-analysis including unpublished data [136] | “When individual patient data from all crossover studies were pooled, persons had 3.60 (95% confidence interval [CI] 2.15, 5.05) fewer cramps in a 4-week period when taking quinine compared with placebo. This compared with an estimate of 8.83 fewer cramps (95% CI 4.16, 13.49) from pooling published studies alone. The corresponding relative risk reductions were 21% (95% CI 12%, 30%) and 43% (95% CI 21%, 65%), respectively” [136]. |  |

*cont’d*

**Table S**2 (cont’d): Examples of reporting bias in the medical literature

| **Indication** | **Intervention** | **Document type, citation** | **Main findings** | **Further reading** |
| --- | --- | --- | --- | --- |
| Wounds | Negative pressure wound therapy | Journal article on a search for unpublished data [144] | “Full-text papers were available on 30% of patients in the 19 completed or discontinued RCTs (495 analysed patients in 10 published RCTs vs. 1154 planned patients in 9 unpublished RCTs). … results data were either not available or requests for results data were not answered; the results of unpublished RCTs could therefore not be considered in the review” [144]. |  |
| Breast enlargement | Silicone implants | Journal article on secrecy in medical research [220] | “Public Citizen sought results from animal studies and consumer complaints relating to the safety of Dow Corning's silicone breast implants. [ref] Although the request specifically excluded product specifications and other manufacturing information, the FDA deemed the information confidential. Dow Corning argued that disclosure could facilitate competitors' safety testing. The court disagreed, characterizing the competitive harm as ’negligible’ and disclosure as ‘unquestionably in the public interest’” [220]. |  |
| ADHD: attention-deficit hyperactivity disorder; AMI: acute myocardial infarction; FDA: Food and Drug Administration; GSK: GlaxoSmithKline; MDD: major depressive disorder; NIH: National Institutes of Health; NPWT: negative pressure wound therapy; RCT: randomized controlled trial; SR: systematic review; SSRI: selective serotonin reuptake inhibitor | | | | |
